# Atypical Presentation of Hepatocellular Carcinoma in a Chronic Alcoholic: Diagnostic Challenges and Therapeutic Approach

**DOI:** 10.7759/cureus.67607

**Published:** 2024-08-23

**Authors:** Bhagyasri Nunna, Pratapsingh Parihar, Suhit Naseri, Rishabh Dhabalia, Saraswathula Bharadwaj

**Affiliations:** 1 Radiodiagnosis, Jawaharlal Nehru Medical College, Datta Meghe Institute of Higher Education & Research, Wardha, IND; 2 Pathology, Jawaharlal Nehru Medical College, Datta Meghe Institute of Higher Education & Research, Wardha, IND

**Keywords:** histopathological evaluation, radiofrequency ablation, diagnostic imaging, alcohol-related liver disease, liver cirrhosis, hepatocellular carcinoma

## Abstract

Hepatocellular carcinoma (HCC) is a primary malignancy of the liver, often arising in the context of chronic liver disease and cirrhosis. This case report describes the clinical presentation, diagnostic evaluation, and therapeutic intervention of a 72-year-old male with a long-standing history of alcohol use who presented with right hypochondrial pain. A 72-year-old male with a 20-year history of alcohol consumption presented with a one-month history of dull, aching pain in the right hypochondrium. Diagnostic imaging, including abdominal ultrasound and contrast-enhanced computed tomography (CECT), revealed significant hepatomegaly with nodular and irregular liver margins, free fluid in the abdomen and pelvis, and multiple hypodense nodules in both liver lobes. One nodule in the right lobe exhibited characteristic imaging features of hepatocellular carcinoma, including peripheral enhancement on the arterial phase and washout on the delayed phase. Histopathological analysis of a biopsy from the suspicious nodule confirmed the diagnosis of hepatocellular carcinoma. The patient was diagnosed with hepatocellular carcinoma based on clinical, radiological, and histopathological findings. He was subsequently scheduled for radiofrequency tumor ablation. This case underscores the importance of comprehensive diagnostic imaging and histopathological evaluation in patients with liver cirrhosis and suspected HCC, particularly in those with a history of chronic alcohol use.

## Introduction

Hepatocellular carcinoma (HCC) is the most common primary malignancy of the liver and is responsible for a significant proportion of cancer-related deaths worldwide. It predominantly occurs in individuals with underlying chronic liver disease, particularly those with cirrhosis, which is often the result of chronic hepatitis B or C infection, non-alcoholic fatty liver disease (NAFLD), or chronic alcohol abuse [1,2]. Chronic alcohol consumption is a well-established risk factor for liver cirrhosis, which is a major precursor for the development of HCC. The pathogenesis of HCC in the context of alcohol-related liver disease is complex, involving repeated cycles of liver cell injury, inflammation, and regeneration, leading to genetic mutations and malignant transformation [3].

Early detection of HCC is critical for improving survival outcomes, as the disease often presents asymptomatically in its early stages and is frequently diagnosed at an advanced stage when curative treatment options are limited [4]. The diagnosis of HCC relies on clinical evaluation, imaging studies, and histopathological confirmation. Imaging modalities such as ultrasound, computed tomography (CT), and magnetic resonance imaging (MRI) play pivotal roles in detecting and characterizing hepatic lesions. Specifically, contrast-enhanced imaging techniques are essential for identifying the characteristic arterial phase hyperenhancement and delayed phase washout seen in HCC [5]. However, the presence of liver cirrhosis can complicate the interpretation of imaging findings due to the coexistence of benign lesions, such as regenerative and dysplastic nodules, which can mimic or obscure the presence of HCC [6].

Given the high risk of HCC in patients with cirrhosis, particularly those with a history of chronic alcohol use, routine surveillance with imaging and serum alpha-fetoprotein (AFP) levels is recommended for early detection [7]. Once diagnosed, the therapeutic approach to HCC depends on the stage of the disease, liver function, and overall patient health. Curative treatments, including surgical resection, liver transplantation, and ablative therapies, are most effective in early-stage HCC, whereas advanced-stage disease may require palliative treatments such as transarterial chemoembolization (TACE) or systemic therapy [8].

## Case presentation

**Figure 1:**
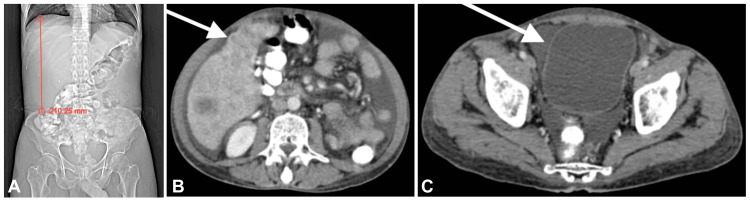
(A) A CT topogram shows an enlarged liver measuring 21 cm, indicative of hepatomegaly. (B) Contrast-enhanced CT (CECT) of the abdomen reveals nodular and irregular liver margins (white arrow), along with free fluid in the abdomen, suggestive of liver cirrhosis and ascites. (C) A CECT of the abdomen demonstrates free fluid in the pelvis (white arrow), suggestive of ascites.

A 72-year-old male presented with a one-month history of dull, aching pain localized in the right hypochondrium. The patient has a 20-year history of alcohol use. Given the complaint of abdominal pain, an abdominal ultrasound was performed, revealing hepatomegaly with multiple isoechoic nodules. One nodule exhibited minimal vascularity on Doppler imaging. Subsequently, a contrast-enhanced computed tomography (CECT) scan of the abdomen and pelvis was conducted, which confirmed an enlarged liver measuring approximately 21 cm, indicative of hepatomegaly (Figure 1A). The liver margins appeared nodular and irregular, with the presence of free fluid in the abdomen (Figure 1B), suggestive of liver cirrhosis. Additionally, the pelvis detected free fluid (Figure 1C), consistent with ascites.

**Figure 2:**
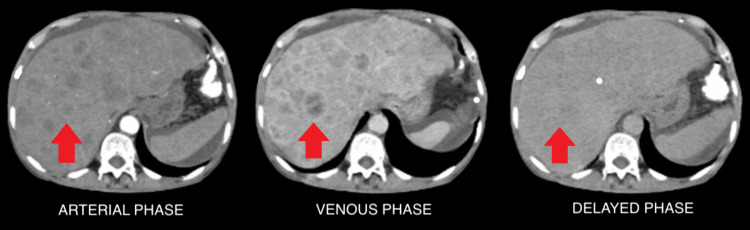
(A–C) A triple-phase CT abdomen shows multiple hypodense nodules showing peripheral enhancement on arterial and venous phases and no washout on the delayed phase in both the lobes of the liver, suggestive of dysplastic nodules.

**Figure 3:**
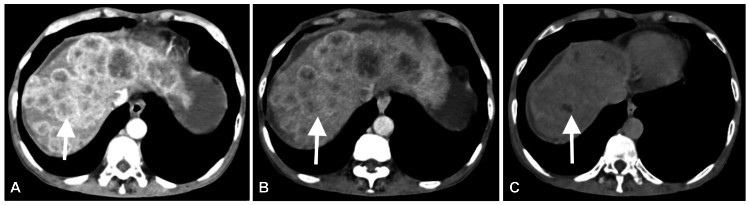
(A) There is one suspicious nodule in the right lobe of the liver (white arrow), showing peripheral enhancement on the arterial phase of the CT abdomen. (B) The suspicious nodule appears isoattenuating on the venous phase of the CT abdomen. (C) The suspicious nodule shows washout and appears hypodense in the delayed phase. These patterns of contrast enhancement on the arterial, venous, and delayed phases of the contrast CT abdomen suggest hepatocellular carcinoma.

The liver demonstrated multiple hypodense nodules showing peripheral enhancement in the arterial and venous phases, with no washout in the delayed phase across both lobes (Figure 2), suggesting dysplastic nodules. One nodule in the right lobe exhibited peripheral enhancement during the arterial phase (Figure 3A), which appeared isoattenuating during the venous phase (Figure 3B), and showed washout, appearing hypodense, during the delayed phase (Figure 3C). These contrast enhancement patterns across arterial, venous, and delayed phases are characteristic of hepatocellular carcinoma.

**Figure 4:**
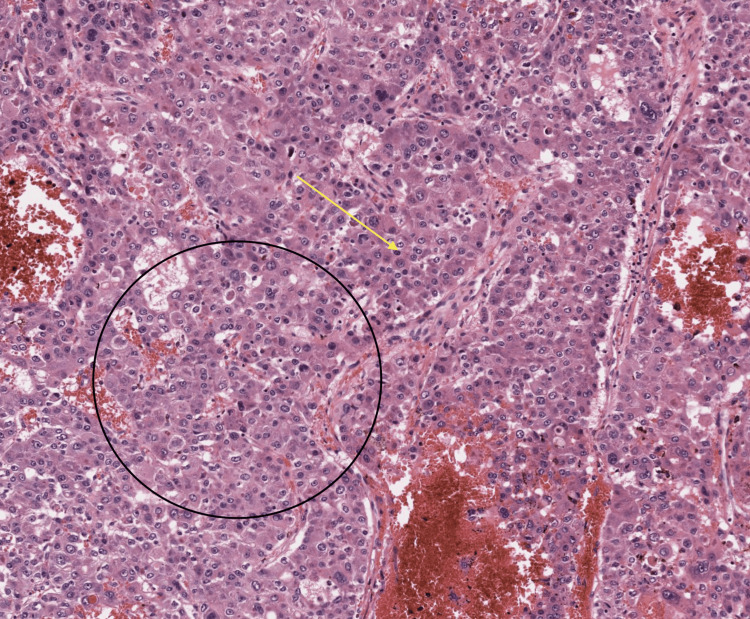
H&E 40× shows tumor cells resembling hepatocytes, which show pleomorphism and form two to eight cell-wide trabeculae, which are separated by sinusoidal spaces. There is a widening of hepatocellular plates, suggestive of hepatocellular carcinoma. H&E: hematoxylin and eosin

Consequently, a biopsy was recommended for the suspicious nodule. Histopathological examination with hematoxylin and eosin (H&E) staining at 40× magnification revealed tumor cells resembling hepatocytes, showing pleomorphism and forming two to eight cell-wide trabeculae separated by sinusoidal spaces. Widening of the hepatocellular plates was also noted (Figure 4), confirming the diagnosis of HCC. Based on the histopathological findings, the patient was scheduled for radiofrequency tumor ablation.

## Discussion

HCC is a prevalent malignancy that often arises in the context of chronic liver disease and cirrhosis, conditions frequently precipitated by chronic alcohol consumption. The insidious onset of HCC and its tendency to develop in a cirrhotic liver make early diagnosis challenging. In this case, the patient's chronic alcohol use, leading to cirrhosis, set the stage for the development of HCC, as evidenced by the radiological and histopathological findings. The association between chronic alcohol consumption and the development of liver cirrhosis is well documented. Alcohol-induced liver injury promotes hepatic fibrosis, eventually leading to cirrhosis, a major risk factor for HCC. According to the European Association for the Study of the Liver (EASL) guidelines, patients with liver cirrhosis, irrespective of etiology, are at an increased risk of developing HCC, and regular surveillance is recommended for early detection of this malignancy [5].

In this patient, the absence of classic symptoms of liver failure or HCC, such as jaundice or significant weight loss, exemplifies the atypical and subtle presentation of HCC in many cases, particularly in those with cirrhosis. Atypical presentations such as isolated right hypochondrial pain can often delay the diagnosis, emphasizing the importance of vigilant surveillance in high-risk patients [9]. Imaging modalities play a pivotal role in the diagnosis of HCC. The characteristic imaging features of HCC on CECT, including arterial phase hyperenhancement and washout in the venous or delayed phases, are critical for the diagnosis, particularly in cirrhotic livers, where lesions can be challenging to differentiate from benign nodules [1]. In this case, the CECT findings raised suspicion for HCC, which was subsequently confirmed by histopathology.

Histopathological confirmation remains the gold standard for HCC diagnosis. The typical histological features of HCC include trabecular patterns of tumor cells resembling hepatocytes, nuclear pleomorphism, and the widening of cell plates, as observed in this case. These findings corroborate the imaging diagnosis and provide a definitive diagnosis [10]. The therapeutic approach for HCC varies depending on the tumor stage, liver function, and overall patient condition. Radiofrequency ablation (RFA) was chosen as the treatment modality for this patient. RFA is a well-established, minimally invasive procedure for early-stage HCC, offering a curative option with a favorable safety profile in patients with cirrhosis [11]. This case highlights the diagnostic challenges posed by the subtle and atypical presentation of HCC in patients with chronic liver disease. It underscores the critical role of comprehensive imaging and histopathological evaluation in accurately diagnosing HCC, particularly in high-risk populations. Early detection through regular surveillance and appropriate therapeutic interventions can significantly improve outcomes for patients with HCC [12].

## Conclusions

In conclusion, this case highlights the clinical presentation and diagnostic evaluation of a 72-year-old male with a history of chronic alcohol use who was diagnosed with HCC. The combination of imaging studies and histopathological examination provided a comprehensive assessment, revealing the presence of dysplastic nodules and hepatocellular carcinoma. The findings underscore the importance of early detection and the role of advanced imaging techniques in accurately diagnosing liver malignancies. Following the diagnosis, the patient was appropriately managed with a plan for radiofrequency tumor ablation, which represents a crucial step in the therapeutic approach for HCC. This case underscores the need for vigilant monitoring and timely intervention in patients with significant risk factors for liver cancer.
